# Compartment Syndrome Secondary to Envenomation by Bothrops asper: Anesthetic Challenges

**DOI:** 10.7759/cureus.105327

**Published:** 2026-03-16

**Authors:** Ashley Martínez Acuña

**Affiliations:** 1 General Medicine, University of Costa Rica, San José, CRI

**Keywords:** bothrops asper, compartment syndrome, perioperative care, snakebite, snake venom

## Abstract

Snakebite envenomation caused by *Bothrops asper* is a significant public health problem in Costa Rica and may result in severe local and systemic complications. A 25-year-old previously healthy female sustained a *Bothrops asper* bite to the right upper limb and subsequently developed extensive edema, muscle necrosis, and clinical compartment syndrome requiring urgent fasciotomy. The clinical course was complicated by venom-induced coagulopathy, ulnar artery thrombosis, anemia requiring transfusion, multiple surgical debridements, negative-pressure wound therapy, and split-thickness skin grafting. From a perioperative perspective, coagulopathy significantly influenced anesthetic planning, favoring general anesthesia over regional or neuraxial techniques to reduce bleeding risk. Postoperative pain management was challenging due to combined nociceptive and neuropathic mechanisms; morphine-induced hallucinations prompted transition to a multimodal, opioid-sparing regimen including pregabalin and transdermal lidocaine. Persistent ulnar and median neuropathy with functional impairment remained, despite rehabilitation. This case highlights the complexity of perioperative management in snakebite-associated compartment syndrome and underscores the importance of individualized anesthetic planning, close hemostatic monitoring, and multimodal analgesia to reduce long-term morbidity.

## Introduction

Snakebite envenomation is a frequent condition in tropical regions and represents a significant public health problem due to its potential to cause severe local and systemic complications. The World Health Organization classifies snakebite as a neglected tropical disease, with an estimated 5.4 million bites occurring globally each year, leading to substantial morbidity and mortality, particularly in rural regions of Asia, Africa, and Latin America [1].

In Costa Rica, snakebite envenomation represents an important public health concern, particularly in rural and agricultural regions where human activity overlaps with snake habitats. The country reports approximately 400-600 snakebite cases annually, with most incidents occurring in tropical areas along the Caribbean and Pacific regions. Victims are predominantly young male agricultural workers, reflecting occupational exposure during farming and outdoor activities. Although mortality rates remain relatively low due to well-established surveillance systems and the availability of antivenom produced by the Instituto Clodomiro Picado, snakebite envenomation continues to cause significant morbidity and long-term functional sequelae [2].

In Central America, particularly in Costa Rica, most clinically significant snakebite envenomations are caused by *Bothrops asper*, commonly known as the terciopelo or fer-de-lance. This species is responsible for the majority of severe snakebites in the region and is associated with greater clinical severity compared with other species. Envenomation by *Bothrops asper* frequently results in complications such as extensive local tissue necrosis, hemorrhage, coagulopathy, compartment syndrome, and permanent functional sequelae including limb disability or amputation [3-5].

*Bothrops asper* belongs to the Viperidae family and the Crotalinae subfamily (pit vipers), characterized by the presence of heat-sensing loreal pits and long solenoglyphous fangs capable of delivering venom deeply into tissues. The venom of this species is a complex mixture of bioactive proteins and enzymes, including snake venom metalloproteinases (SVMPs), phospholipases A₂ (PLA₂), serine proteases, and other toxins that contribute to local tissue destruction, hemorrhage, edema, and systemic coagulopathy. These mechanisms explain the significant local damage and systemic complications frequently observed in *Bothrops asper* envenomations [6-8].

Among the local complications, compartment syndrome secondary to snakebite is uncommon but may irreversibly compromise limb function if not recognized and treated promptly [9,10]. From a perioperative perspective, these cases present significant challenges related to venom-induced hemostatic instability, the need for repeated surgical interventions, and complex pain management [11].

This case report describes the perioperative, anesthetic, and analgesic challenges associated with *Bothrops asper* envenomation complicated by compartment syndrome.

## Case presentation

**Figure 1:**
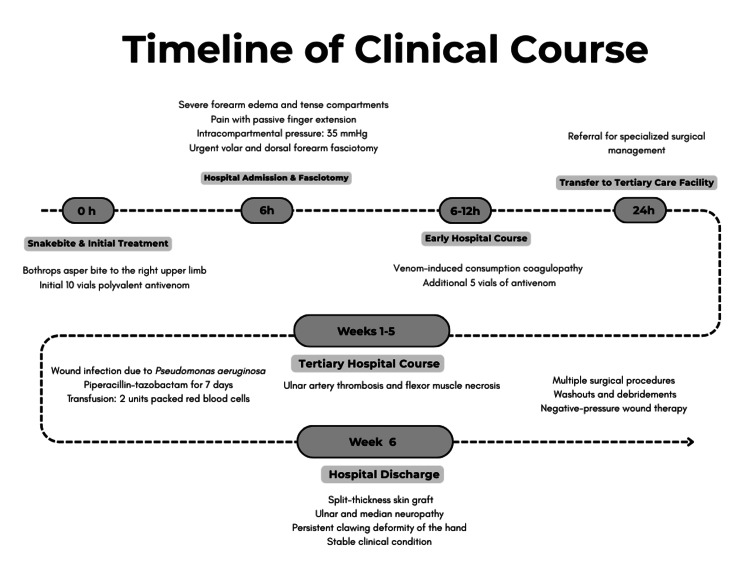
Timeline of the clinical course following Bothrops asper envenomation A timeline summarizing the clinical course of the patient from the time of envenomation through hospitalization.

A 25-year-old previously healthy woman sustained a bite from *Bothrops asper* to the right upper limb during university activities in Limón Province, Costa Rica. She initially received polyvalent anti-viperid antivenom (PoliVal-ICP®, Instituto Clodomiro Picado, Costa Rica) at a primary care center. An initial dose of 10 vials was administered intravenously after reconstitution and dilution in normal saline according to institutional protocols for moderate to severe envenomation. Tetanus immunization status was assessed, and prophylaxis was administered according to institutional protocols. Symptoms progressed rapidly after envenomation, prompting referral to a regional hospital for further management (Figure 1).

**Figure 2:**
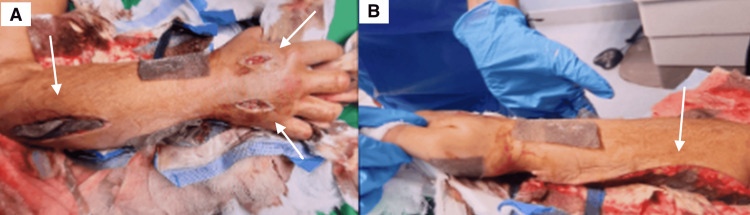
Forearm fasciotomy for compartment decompression (A) Intraoperative photograph demonstrating fasciotomy incision sites (white arrows) used to decompress the forearm compartments. (B) An extended fasciotomy incision (white arrow) exposing the compartment region after surgical decompression.

On admission, extensive edema and areas of necrosis involving the extensor and deep flexor compartments of the forearm were documented. The affected limb showed marked swelling with tense compartments and severe pain with passive extension of the fingers. The patient also reported severe pain disproportionate to the physical findings and progressive paresthesia in the affected hand, further raising clinical suspicion for acute compartment syndrome. Intracompartmental pressure measurements were obtained using a handheld compartment pressure monitoring device and demonstrated pressures of 35 mmHg in the volar compartment, exceeding the commonly accepted diagnostic threshold of ≥30 mmHg for acute compartment syndrome. Based on these objective findings, urgent volar and dorsal forearm fasciotomy was performed to prevent further neurovascular compromise. Although severe local manifestations of *Bothrops asper* envenomation may mimic compartment syndrome due to marked edema and inflammation, the elevated intracompartmental pressures in this case supported the diagnosis and the need for surgical decompression. Intraoperative findings demonstrated extensive soft tissue compromise and compartment release following fasciotomy (Figure 2).

During hospitalization, the patient developed laboratory evidence of venom-induced consumption coagulopathy, including prolonged prothrombin time and decreased fibrinogen levels. Additional antivenom therapy consisting of five vials was administered along with fluid replacement. Due to the complexity of her condition, the patient was transferred to a tertiary care facility, where she remained hemodynamically stable and required multiple surgical procedures, including wound washouts, debridements, and negative-pressure wound therapy. Serial laboratory monitoring during the first two weeks of hospitalization demonstrated progressive improvement of coagulation parameters following treatment. However, the patient developed progressive anemia with a nadir hemoglobin level of 6.8 g/dL on hospital day 7, which required transfusion of two units of packed red blood cells. Subsequent laboratory values demonstrated gradual hematologic recovery, and renal function remained stable throughout hospitalization. Serial laboratory parameters during the first two weeks are summarized in Table 1.

**Table 1:** Serial laboratory parameters during the first two weeks of hospitalization Laboratory values were monitored to assess the progression of venom-induced consumption coagulopathy and systemic effects of envenomation. Initial abnormalities included prolonged prothrombin time and decreased fibrinogen levels, which gradually improved after antivenom therapy and supportive care. Hemoglobin reached a nadir of 6.8 g/dL on hospital day 7, requiring transfusion of two units of packed red blood cells, followed by progressive hematologic recovery.

Parameter	Admission	Day 1	Day 3	Day 7	Day 10	Day 14
Hemoglobin (g/dL)	11.2	10.6	9.4	6.8	8.9	10.5
Platelets (×10³/µL)	165	150	138	182	215	248
PT (seconds)	18.5	20.1	17.2	14.9	13.5	12.8
aPTT (seconds)	42	45	39	34	31	29
Fibrinogen (mg/dL)	140	120	165	220	275	310
Creatinine (mg/dL)	0.9	0.9	0.8	0.8	0.8	0.8
CK (U/L)	780	1250	980	620	340	180

**Figure 3:**
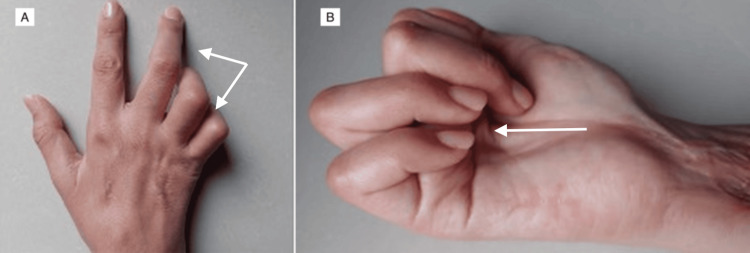
Late postoperative functional deficit associated with ulnar neuropathy (A) Resting hand position demonstrating a clawing pattern of the fourth and fifth digits, consistent with ulnar claw deformity. (B) Attempted finger flexion demonstrating motor deficit with impaired flexion of the affected digits, indicating ulnar nerve dysfunction.

During one intervention, thrombosis of the ulnar artery and complete necrosis of the flexor digitorum superficialis and flexor carpi ulnaris muscles were identified, necessitating extensive muscle resections. Although routine antibiotic prophylaxis is not universally recommended following snakebite envenomation, antimicrobial therapy may be indicated in cases with extensive tissue necrosis, surgical intervention, or clinical concern for infection. In this case, empiric antibiotic therapy with piperacillin-tazobactam (4.5 g intravenously every six hours) was initiated due to extensive soft-tissue necrosis and the need for repeated surgical procedures. Wound cultures obtained during surgical debridement at the tertiary care facility grew Pseudomonas aeruginosa, confirming a secondary wound infection and supporting the indication for broad-spectrum antimicrobial therapy. Antibiotic treatment was continued for seven days, with clinical improvement and no evidence of systemic infectious complications. The patient remained hospitalized for approximately six weeks, during which she underwent multiple wound care procedures, repeated negative-pressure wound therapy changes, and ultimately split-thickness skin grafting of the right forearm. During postoperative pain management, morphine was initially administered but was associated with visual hallucinations, requiring adjustment to a multimodal opioid-sparing analgesic regimen. During recovery, neuropathy involving the ulnar and median nerves became evident, resulting in functional impairment of the affected limb. Treatment with pregabalin was initiated as an adjuvant for neuropathic pain, along with transdermal lidocaine patches and rehabilitation therapy aimed at improving range of motion, function, and pain control. Despite partial improvement, distal motor sequelae persisted. Persistent motor deficit with clawing deformity of the hand secondary to ulnar neuropathy is shown in Figure 3.

## Discussion

*Bothrops asper* envenomation complicated by compartment syndrome represents a highly complex perioperative scenario in which systemic venom effects, progressive tissue damage, and the need for repeated surgical interventions converge [7]. The management of suspected compartment syndrome following snakebite remains controversial in the toxicology literature. Venom-induced edema may produce elevated intracompartmental pressures that improve after adequate antivenom administration; therefore, antivenom therapy is considered the cornerstone of treatment in these cases [8]. However, progressive swelling, severe pain, and clinical findings concerning for compartment syndrome may develop despite appropriate medical management. Although distal pulses may remain palpable, the presence of intact pulses does not exclude compartment syndrome, as arterial flow may persist even in the setting of critically elevated compartment pressures. In such situations, when clinical deterioration persists and there is concern for impending neurovascular compromise, surgical decompression with fasciotomy may be required to prevent irreversible tissue injury.

Venom-induced coagulopathy is multifactorial and may persist or fluctuate even after appropriate antivenom administration [9-11]. In this context, hemostatic instability becomes a critical determinant of anesthetic management. In the present case, evolving coagulation abnormalities required continuous reassessment prior to each surgical intervention. The potential risk of bleeding ruled out neuraxial anesthesia and deep regional techniques, favoring general anesthesia as the safest option [12]. This case underscores the importance of dynamic laboratory monitoring and individualized anesthetic planning in patients with venom-related coagulopathy, even when hemodynamic parameters remain stable.

Pain management in snakebite envenomation is frequently complex due to the coexistence of nociceptive, inflammatory, ischemic, and neuropathic mechanisms [13]. In addition to direct venom-mediated tissue injury, repeated surgical trauma and neurovascular compromise further amplify pain perception. In this patient, morphine administration was associated with visual hallucinations, a recognized but uncommon adverse effect of opioid therapy [14]. The transition to a multimodal, opioid-sparing regimen incorporating pregabalin and transdermal lidocaine improved tolerability while targeting neuropathic components of pain. This highlights the value of multimodal analgesia in complex perioperative cases where single-agent opioid therapy may be insufficient or poorly tolerated [15].

The development of persistent ulnar and median neuropathy illustrates the severity of local tissue destruction associated with Viperidae envenomation. Despite timely surgical and medical management, progressive edema, ischemia, and muscle necrosis can result in irreversible neurologic deficits [17]. This emphasizes that clinical progression may occur even after initial antivenom therapy, reinforcing the need for vigilant follow-up and early rehabilitation strategies to minimize chronic disability.

Overall, this case demonstrates that severe *Bothrops asper* envenomation extends beyond acute toxicity and requires coordinated multidisciplinary management, structured perioperative risk assessment, and comprehensive pain control strategies to optimize functional outcomes.

## Conclusions

*Bothrops asper* envenomation complicated by compartment syndrome represents a rare but severe clinical entity requiring early recognition and coordinated multidisciplinary management. Venom-induced coagulopathy significantly influences perioperative decision-making, often limiting the use of regional or neuraxial anesthesia and necessitating individualized anesthetic planning based on dynamic hemostatic assessment.

Furthermore, snakebite-related tissue injury frequently results in complex pain syndromes with both nociceptive and neuropathic components. An opioid-sparing, multimodal analgesic strategy may improve safety and tolerability in patients undergoing repeated surgical interventions. Despite appropriate medical and surgical treatment, long-term neurologic sequelae may persist, underscoring the importance of early rehabilitation and structured follow-up to optimize functional recovery.
